# Structural Coupling of Extrinsic Proteins with the Oxygen-Evolving Center in Photosystem II

**DOI:** 10.3389/fpls.2016.00084

**Published:** 2016-02-05

**Authors:** Kentaro Ifuku, Takumi Noguchi

**Affiliations:** ^1^Graduate School of Biostudies, Kyoto UniversityKyoto, Japan; ^2^Graduate School of Science, Nagoya UniversityAichi, Japan

**Keywords:** extrinsic proteins, FTIR, oxygen-evolving complex, photosystem II, photosynthetic electron transport

## Abstract

Photosystem II (PSII), which catalyzes photosynthetic water oxidation, is composed of more than 20 subunits, including membrane-intrinsic and -extrinsic proteins. The PSII extrinsic proteins shield the catalytic Mn_4_CaO_5_ cluster from the outside bulk solution and enhance binding of inorganic cofactors, such as Ca^2+^ and Cl^-^, in the oxygen-evolving center (OEC) of PSII. Among PSII extrinsic proteins, PsbO is commonly found in all oxygenic organisms, while PsbP and PsbQ are specific to higher plants and green algae, and PsbU, PsbV, CyanoQ, and CyanoP exist in cyanobacteria. In addition, red algae and diatoms have unique PSII extrinsic proteins, such as PsbQ′ and Psb31, suggesting functional divergence during evolution. Recent studies with reconstitution experiments combined with Fourier transform infrared spectroscopy have revealed how the individual PSII extrinsic proteins affect the structure and function of the OEC in different organisms. In this review, we summarize our recent results and discuss changes that have occurred in the structural coupling of extrinsic proteins with the OEC during evolutionary history.

## Introduction

Photosystem II (PSII) is a key protein complex involved in light-energy conversion reactions in photosynthesis. PSII converts light energy into the electrochemical potential energy required to split water into H^+^, electrons, and molecular oxygen (Debus, 1992). The PSII complex is composed of more than 20 subunits, with CP47, CP43, D1, D2, Cyt *b*_559_ α- and β-subunits, and PsbI comprising the reaction center complex (Satoh, 2008). In addition, a number of small peripheral subunits stabilize the PSII core (Pagliano et al., 2013). Recent X-ray structural analysis of the cyanobacterial PSII complex has revealed the location of most subunits, pigments, and redox cofactors (Ferreira et al., 2004; Guskov et al., 2009; Umena et al., 2011; Suga et al., 2015). In PSII, light excitation of the primary donor P680, comprising a special pair of chlorophyll (Chl) *a*, results in electron transfer to a nearby pheophytin, followed by electron transfer to the acceptor quinones (Q_A_ and Q_B_). The resulting cation radical of P680^+^ receives electrons via a redox-active tyrosine of D1, Y_Z_, from the Mn_4_CaO_5_ cluster. The Mn_4_CaO_5_ cluster converts two water molecules into one molecular oxygen and four protons through a light-driven cycle consisting of five intermediates called S*_i_* states (*i* = 0–4; McEvoy and Brudvig, 2006). Among them, the S_1_ state is the most dark-stable, and flash illumination advances each S*_i_* state (*i* = 0–3) to the next S*_i+1_* state. Molecular oxygen is released during the S_3_–S_4_–S_0_ transition after the transient S_4_ state (Vinyard et al., 2013).

**FIGURE 1:**
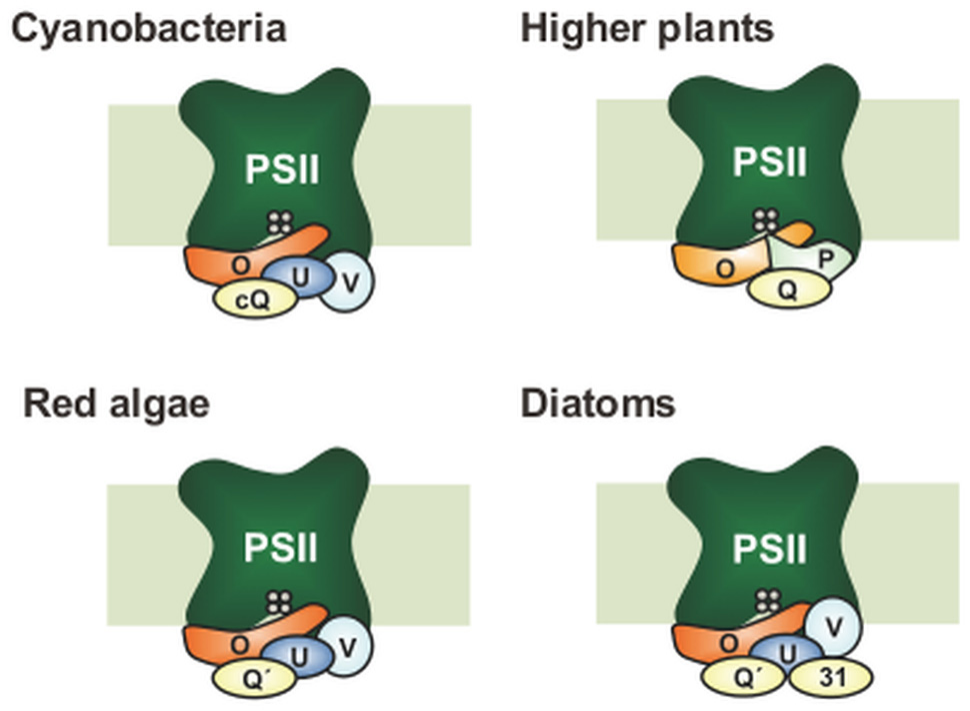
**Differences in subunit composition of the extrinsic proteins of photosystem II (PSII)**. Only PsbO (O) has remained in all photosynthetic organisms. In higher plants, PsbV (V) and PsbU (U) were lost, while PsbP (P) and PsbQ (Q) have been derived from CyanoP (cP) and CyanoQ (cQ), respectively. Green algal PSII is basically similar to that in higher plants, whereas green-algal PsbQ is more similar to PsbQ′ (not shown). CyanoP has been excluded from the model, because its stable association with PSII is still unclear. In red algae and diatoms, PsbQ′ (Q′) has developed from CyanoQ; in addition, Psb31 (31) is found in diatoms. These models do not show the exact location and interaction of the PSII extrinsic subunits.

The mechanism of water oxidation and the subunit structure of the PSII core are highly conserved across oxygenic photosynthetic organisms ranging from cyanobacteria to flowering plants, while the composition of the extrinsic subunits of PSII surrounding the catalytic Mn_4_CaO_5_ cluster has undergone a large evolutionary change (Bricker et al., 2012; Ifuku, 2015; **Figure 1**). Green eukaryotes including higher plants and green algae have a set of three extrinsic proteins — PsbO, PsbP, and PsbQ — that bind to the lumenal surface of PSII. In cyanobacterial PSII, PsbV, and PsbU are present instead of PsbP and PsbQ (Shen and Inoue, 1993). Furthermore, cyanobacteria have PsbP and PsbQ homologs (CyanoP and CyanoQ, respectively; Kashino et al., 2002; Thornton et al., 2004), but these proteins are not yet included in the current crystal structures. Furthermore, there are multiple homologs of PsbP and PsbQ in the chloroplast thylakoid lumen and some of them play important roles in the assembly and stability of various thylakoid membrane complexes including PSII, PSI, and the chloroplast NADH dehydrogenase-like complex (Ifuku et al., 2008, 2010, 2011a; Bricker et al., 2013; Ifuku, 2014). These facts suggest that PsbP and PsbQ proteins in green plants have evolved from their cyanobacterial homologs, where considerable genetic and functional modifications have occurred to generate the present eukaryotic forms.

In red algae and diatoms, PsbQ′, a 20-kDa homolog of CyanoQ, is bound to PSII as an extrinsic subunit in addition to PsbO, PsbU, and PsbV (Ohta et al., 2003). Diatoms possess an additional specific extrinsic subunit, Psb31 (Okumura et al., 2008), and recent structural analysis suggests that Psb31 might be a homolog of PsbQ (Nagao et al., 2013). Although high-resolution structures of individual PSII extrinsic subunits have been reported for eukaryotes (Calderone et al., 2003; Ifuku et al., 2004; Balsera et al., 2005; Kopecky et al., 2012; Cao et al., 2015), their binding sites and topologies have not been determined because crystallographic information derived from prokaryotic cyanobacterial PSII cannot be fully applied to eukaryotic PSII.

A number of reviews have been published about function, structure, and evolution of PSII extrinsic proteins in various photosynthetic organisms (Roose et al., 2007b; Enami et al., 2008; Ifuku et al., 2008, 2011b; Bricker and Frankel, 2011; Bricker et al., 2012; Ifuku, 2014, 2015). In this review, we focus on the structural coupling of extrinsic proteins with the oxygen-evolving center (OEC) in different photosynthetic organisms, which has been investigated by reconstitution experiments combined with light-induced Fourier transform infrared (FTIR) difference spectroscopy (Tomita et al., 2009; Ido et al., 2012; Uno et al., 2013; Nishimura et al., 2014; Nagao et al., 2015). This technique can detect the structural changes in the OEC in the S-state transitions, including the changes in the secondary structures of polypeptide main chains, amino acid side chains, and hydrogen bond networks of proteins and water molecules (Noguchi, 2007, 2015; Debus, 2015). The results of FTIR are evaluated in light of the current knowledge on the subunit interactions in PSII and also discussed in terms of the changes that occurred during evolution.

## Higher Plants

### Functions of Extrinsic Proteins in PSII, Briefly

The molecular functions of the extrinsic proteins in higher plants have been intensively analyzed by release-reconstitution experiments using PSII-enriched membrane preparations (BBY membranes; Berthold et al., 1981). Briefly, PsbO is most strongly bound to PSII and stabilizes the Mn cluster (Kuwabara et al., 1985). PsbP is involved in Ca^2+^ and Cl^-^ retention in PSII (Ghanotakis et al., 1984a), and PsbQ participates primarily in Cl^-^ retention (Akabori et al., 1984; Miyao and Murata, 1985). In addition, PsbP and PsbQ have a role in protecting the Mn cluster from reductants in the bulk solution (Ghanotakis et al., 1984b). It is reasonable that PsbO, commonly found in PSII in all oxygenic photosynthetic organisms, is essential for the accumulation and assembly of PSII *in planta* (Murakami et al., 2002; Yi et al., 2005). In addition, genetic studies using knockdown or knockout plants have suggested that PsbP, but not PsbQ, is essential for the oxygen-evolving activity of PSII *in vivo* (Ifuku et al., 2005b; Yi et al., 2007), suggesting that the development of the PsbP protein as an extrinsic subunit would be the crucial event for PSII function during evolution (Ido et al., 2009; Ifuku et al., 2011b). PsbQ is reported to be required for PSII stability under prolonged low-light conditions (Yi et al., 2006).

It was suggested that PsbP and PsbQ also play an important role in stabilizing the architecture of the PSII-light-harvesting complex (LHC) II supercomplexes in higher plants (Ifuku et al., 2011b). PsbP knockdown by RNAi caused a severe decrease in the levels of PSII-LHCII supercomplexes, whereas the amounts of unattached LHCII trimers and minor LHCs were significantly increased (Ido et al., 2009). Similarly, the abundance of PSII-LHCII supercomplexes decreased in mutants lacking PsbQ and/or PsbR (Allahverdiyeva et al., 2013). PsbR is another subunit, mostly extrinsic and specifically found in green plants, which stabilizes the binding of PsbP (Suorsa et al., 2006; Allahverdiyeva et al., 2007; Liu et al., 2009). Furthermore, depletion of PsbQ and/or PsbR affects short-term regulatory mechanisms such as state transitions and non-photochemical quenching (Allahverdiyeva et al., 2013). These observations are relevant to the chemical-crosslinking study showing the interaction of PsbP and PsbQ with a minor antenna protein CP26 and the inner core antenna CP43 (Ido et al., 2014).

### Structural Coupling with the OEC

**FIGURE 2:**
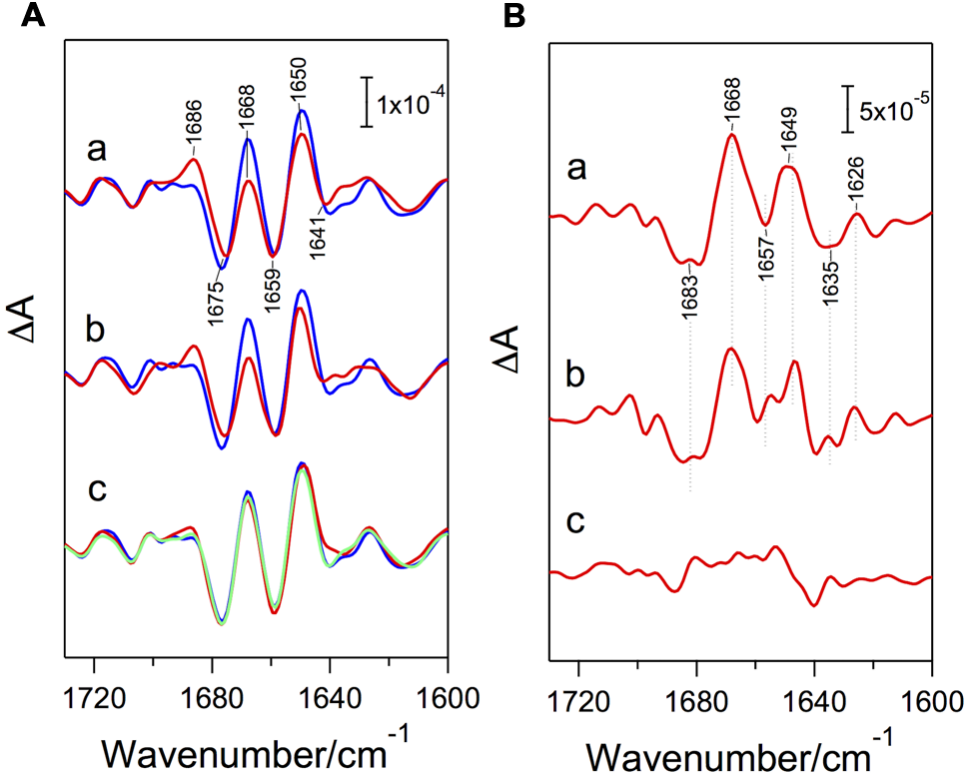
**(A)** Amide I region of the S_2_-minus-S_1_ FTIR difference spectra (red lines) of NaCl-washed (a), CaCl_2_-washed (b), and NaCl-washed, PsbP-reconstituted (c) PSII membranes in comparison with the spectrum of untreated PSII membranes (blue lines). A green line in (c) is the spectrum of PSII reconstituted with ^13^C-labeled PsbP. **(B)** Double-difference spectra between the S_2_-minus-S_1_ spectra of treated and untreated PSII samples. (a) untreated-*minus*-NaCl-washed PSII; (b) untreated-*minus*-CaCl_2_-washed PSII; (c) untreated-*minus*-NaCl-washed, PsbP-reconstituted PSII. Reproduced from Tomita et al. (2009).

The importance of PsbP in structural coupling with the OEC has been suggested by *in vitro* reconstitution studies combined with FTIR measurements (Tomita et al., 2009). PSII membranes depleted of PsbP and PsbQ by NaCl washing showed clear changes in amide I bands (1700-1600 cm^-1^; C=O stretches of backbone amides), which reflect structural changes in polypeptide main chains, in S_2_-minus-S_1_ FTIR difference spectra (**Figure 2**), whereas no appreciable changes were observed in the bands of carboxylate and imidazole groups, which arise from ligands or the immediate surroundings of the Mn_4_CaO_5_ cluster. Further depletion of PsbO by CaCl_2_ washing did not induce further changes (**Figure 2**). The original amide I features were recovered by reconstitution of the NaCl-washed PSII membranes with a recombinant PsbP heterologously expressed in *Escherichia coli*, and the same recovery was observed with ^13^C-labeled PsbP (Tomita et al., 2009). These results indicate that the PsbP protein, but not PsbQ or PsbO, affects the protein conformation around the Mn_4_CaO_5_ cluster in the intrinsic proteins without changing the immediate interactions of the Mn_4_CaO_5_ cluster in the OEC.

It should be noted that controversial data have recently been reported stating that the removal of PsbP and PsbQ from the PSII core preparations of spinach showed no significant effect on the S_2_-minus-S_1_ spectrum (Offenbacher et al., 2013). However, the control spectrum in this report showed an amide I feature typical of PsbP-depleted PSII: lower and higher intensities at the 1668 and 1686 cm^-1^ peaks, respectively (**Figure 2**). We thus speculate that the control PSII sample was actually depleted of PsbP and PsbQ during FTIR measurement, possibly by the presence of potassium ferricyanide in addition to 20 mM CaCl_2_.

**FIGURE 3:**
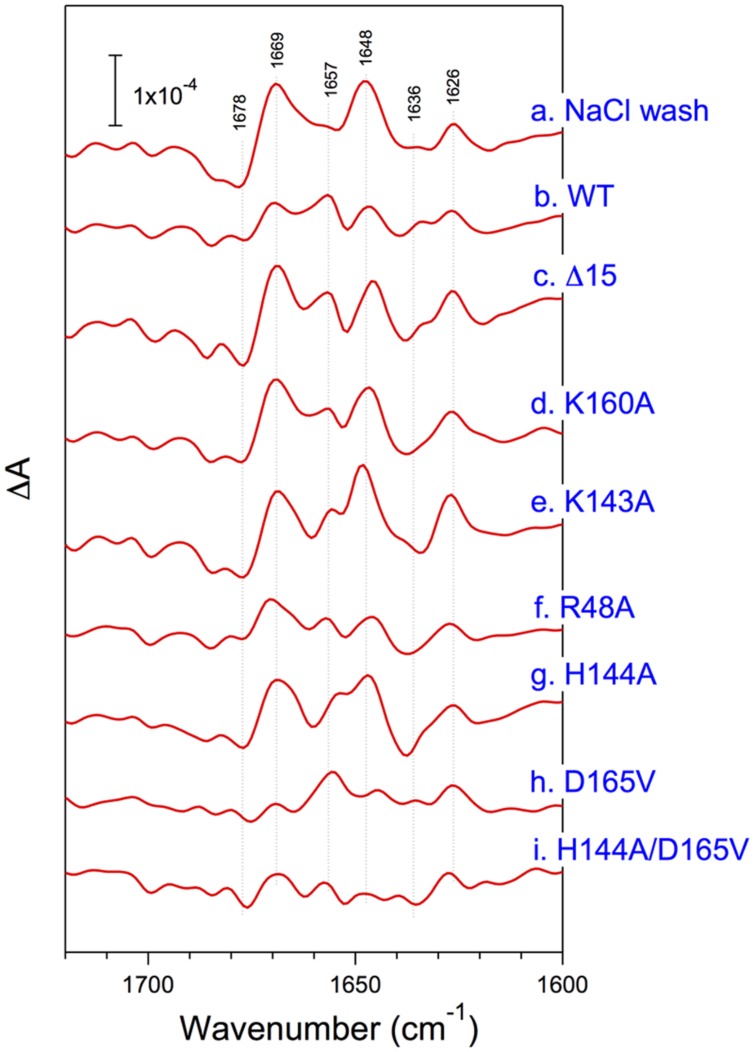
**Amide I region of untreated-minus-treated double difference spectra of the S_2_Q_A_^-^-minus-S_1_Q_A_ FTIR spectra of PSII membranes from spinach**. PSII membranes were NaCl-washed (a) and then reconstituted with WT- (b), Δ15- (c), K160A- (d), K142A- (e), R48A- (f), H144A- (g), D165V- (h), and H144A/D165V- (i) PsbP. Reproduced from Ido et al. (2012), Kakiuchi et al. (2012), and Nishimura et al. (2014).

**FIGURE 4:**
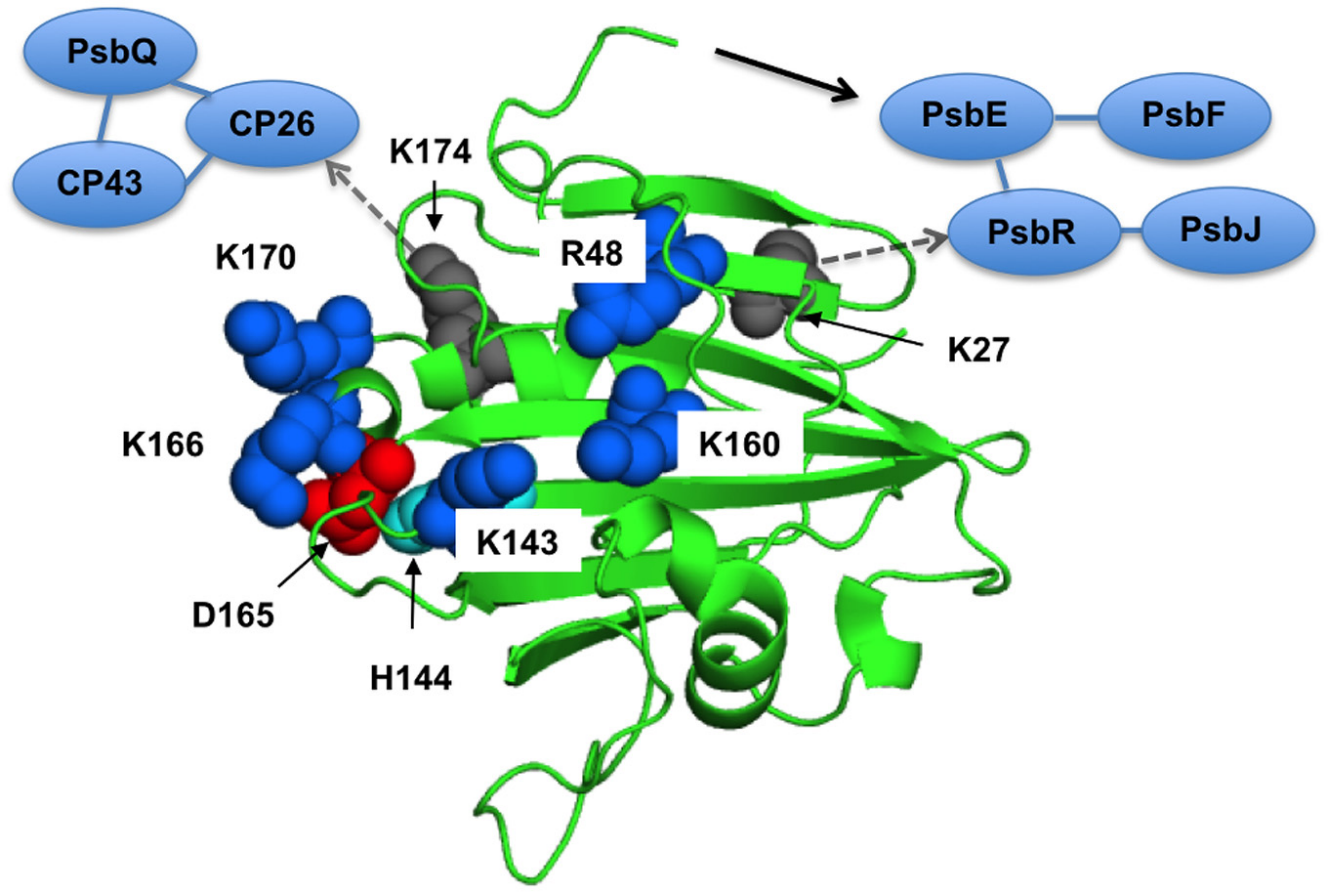
**The proposed interaction surface of the extrinsic PsbP protein with the PSII core**. The basic amino-acid residues important for the structural coupling with the OEC are labeled and shown as blue sphere models in a spinach PsbP crystal structure (PDB ID: 4RTI, Cao et al., 2015). Asp-165 and His-144, shown as red and cyan spheres, make a salt bridge required for the proper interaction with PSII (Ido et al., 2012). Lys-27 and Lys-174, shown as gray spheres, are suggested to interact with PsbR and CP26, respectively (Ido et al., 2014). A black arrow indicates the N-terminal sequence of PsbP extended to interact with PsbE. Interactions around CP26 and PsbR are also schematically indicated. See text for detail.

The structural coupling of PsbP with the OEC has been studied by truncation and site-directed mutagenesis. **Table 1** summarizes the results of our reconstitution-FTIR experiments, while **Figure 3** shows the amide I region of the FTIR double difference (untreated-minus-treated) spectra. The positions of mutated amino-acid residues are indicated in the cartoon model of the spinach PsbP structure reported recently (Cao et al., 2015; (**Figure 4**). Overall, the abilities of mutated PsbPs to restore the structural changes around the OEC correlate well with the oxygen-evolving activity reconstituted by each PsbP species. This strongly suggests the tight structural coupling of PsbP with the OEC in higher plant PSII.

**Table 1:** Effects of various PsbP mutations on structural coupling with the oxygen-evolving center (OEC).

PsbP	^a^O_2_ activity	^b^Binding to PSII	^c^FTIR	Reference
Wild-type (spinach)	++++	++++	◯	Tomita et al., 2009
Δ9	++	+++	n.d.	Miyao et al., 1988;
				Ifuku et al., 2008
Δ15	–	–	×	Ifuku et al., 2005a;
				Tomita et al., 2009;
				Kakiuchi et al., 2012
Δ19	–	–	n.d.	Ifuku and Sato, 2002
K160A	++	+++	×	Nishimura et al., 2014
K143A	++	+++	×	
R48A	++	+++	△	
K143A/R48A	+	+	n.d.	
K143A/K160A/R48A	–	–	n.d.	
K11A	++++	+++	n.d.	
K13A	++++	+++	n.d.	
H144A	+	++++	×	Ido et al., 2012
D165V	++++	++++	◯	
E177V	++++	++++	n.d.	
H144A/D165V	++++	++++	◯	

*^a^O_2_-evolving activities were measured in buffer depleted of Ca^2+^ and Cl^-^. The activity restored by binding of mutant PsbP is indicated by the number of plus signs (+). A minus sign (-) indicates no recovery. ^b^Binding to PSII was estimated by imaging SDS-PAGE gels. The binding affinity of mutant PsbP to PSII is indicated by the number of plus signs (+). A minus sign (-) indicates no binding to PSII. ^c^Recovery of the amide I bands in the S_2_-minus-S_1_ FTIR difference spectra, which were measured in the presence of Ca^2+^ and Cl^-^. ◯, full recovery; △, partial recovery;×, no recovery; n.d., not determined*.

Noticeably, reconstitution with Δ15-PsbP, in which the 15 N-terminal residues were truncated, did not restore the amide I bands, indicating that the interaction of the N-terminal region would be responsible for inducing the conformational changes around the OEC (Tomita et al., 2009). In fact, Δ15-PsbP did not restore the Ca^2+^ and Cl^-^ retention ability upon rebinding to PSII (Ifuku and Sato, 2002). It was found that additional binding of PsbQ partly restores the ability of Δ15-PsbP to induce the proper conformational changes and activate oxygen evolution in the OEC (Kakiuchi et al., 2012). These facts suggest that the N-terminal region of PsbP is not essential but may have a function in recruiting PsbP to the proper binding site to induce the conformational changes. PsbQ would have an auxiliary role in supporting PsbP binding and function in higher plants.

The central αβα structure of PsbP in its C-terminal domain is also important for the interaction with PSII to induce the conformational change in the OEC. The PsbP surface has a basic region consisting of conserved Arg48, Lys143, and Lys160, and the mutations of R48A, K143A, and K160A result in lower binding affinity with PSII, and double and triple mutation of those residues severely diminishes their binding to PSII (Nishimura et al., 2014). Even when a saturating amount of protein is used for the reconstitution, the R48A, K143A, and K160A proteins cannot restore the rate of oxygen evolution fully at low chloride concentrations. The results of FTIR were consistent with the above finding, showing that these mutated proteins are not able to induce the normal conformational change around the Mn cluster during the S_1_-to-S_2_ transition. The above observations suggest that the basic surface of PsbP is involved in the electrostatic interaction with the PSII complex. This binding topology of PsbP is further supported by a recent study using synchrotron radiolysis of water to further define buried surface regions by modification with OH^⋅^ (Mummadisetti et al., 2014).

We also investigated the role of the structure around His-144 and Asp-165 in the C-terminal domain of PsbP, which is suggested to be a metal binding site in PsbP (Ido et al., 2012). PsbP with an H144A mutation shows a reduced ability to retain Cl^-^ anions in PSII, whereas the D165V mutation does not affect PsbP function. In FTIR, PsbP-H144A could not restore proper interaction with PSII inducing the conformational change around the Mn cluster during the S_1_-to-S_2_ transition. Consistently, mutations of K166A and K170A near His144 are suggested to affect the PsbP binding to PSII (Tohri et al., 2004). Unexpectedly, the H144A/D165V double mutation suppresses the defect caused by H144A mutation. Presumably, His-144 and Asp-165 form a salt bridge and H144A mutation would disrupt this bridge and liberate Asp-165, inhibiting the proper PsbP-PSII interaction. These residues therefore have a role other than metal binding. The recently observed crystal structure of spinach PsbP suggests an additional binding site for Mn^2+^ coordinated by Asp-98 (Cao et al., 2015). The authors speculated that Mn-binding in this site may induce conformational change of PsbP during the PSII damage–repair cycle. However, PsbPs of some plant species, such as *Arabidopsis* and cucumber, have Ala-98 instead of Asp-98. To discuss the physiological importance of the metal binding site observed in the crystal structures, it is necessary to evaluate the exact metal binding constant for each binding site. Further characterization of mutated PsbP proteins *in vivo* will provide conclusive results.

### Interaction within the PSII Supercomplex

The historical model of the organization of the extrinsic subunits in higher plants is that PsbO binds directly to the PSII core, followed by PsbP binding to PsbO, and then PsbQ binding to PsbP or PsbO. However, recent studies using chemical cross-linking combined with mass spectrometry suggest the direct interactions of PsbP and PsbQ with membrane-intrinsic PSII subunits. Using the zero-length cross-linker 1-ethyl-3-(3-diethylaminopropyl) carbodiimide (EDC), interactions between PsbP and PsbE and between PsbP and PsbR were detected (Ido et al., 2012, 2014). In addition, PsbP and PsbQ were further linked to the CP26 and CP43 light-harvesting proteins. Furthermore, the cross-linked sites between PsbP:Ala-1 and PsbE:Glu-57, PsbP:Lys-27 and PsbR:Asp-22, and PsbP:Lys-174 and CP26:Glu-96 were identified by tandem mass spectrometry. The above information allows us to evaluate the binding manner of extrinsic proteins in the PSII supercomplex in higher plants (Ido et al., 2014) in light of the FTIR studies.

The possible candidates for the polypeptide chains affected by PsbP during the S_1_ to S_2_ transition are those interacting with the Mn_4_CaO_5_ cluster and Cl^-^ ions via ligation or direct hydrogen bonding. In the cyanobacterial PSII core complex, the Mn_4_CaO_5_ cluster and one Cl^-^ ion interact with a number of residues in the C-terminal region of the D1 protein, as well as the Glu-354 and Arg-357 of the CP43 proteins (Umena et al., 2011). In addition, another Cl^-^ ion is coordinated by Lys-317 on the D2 protein (Kawakami et al., 2009). These two Cl^-^ bindings are suggested to be important for the coordination structure of the Mn_4_CaO_5_ cluster and/or for proposed proton channels, thereby keeping the OEC fully active (Pokhrel et al., 2013; Suzuki et al., 2013). Our cross-linking study suggested that PsbP may change the structure of CP43 to induce structural changes around the OEC (Ido et al., 2014). Alternatively, interaction of the N-terminal region of PsbP with PsbE and PsbR, and possibly PsbJ, may affect the structure of D2 by inducing changes around the Cl^-^ binding site. This binding manner of PsbP may be similar to that of PsbV in cyanobacterial systems, as has been suggested in recent reviews (Bricker et al., 2015; Ifuku, 2015).

It should be noted that a later study using a cross-linker bis-sulfosuccinimidyl suberate (BS3), which has a spacer length of 11.4 Å, has proposed another binding model of PsbP and PsbQ in higher plants’ PSII (Mummadisetti et al., 2014). Cross-linking by BS3 failed to detect an interaction between extrinsic and intrinsic PSII proteins, while it indicated that the N-terminal 15-amino-acid residue domain of PsbP was in close proximity (≤11.4 Å) to the C-terminal region of PsbP. Assuming that PsbP and PsbQ are located near the interface between CP43 and CP26, the N-terminal sequence of PsbP should extend toward PsbE (Ido et al., 2012, 2014); however, this model was unsupported by BS3 crosslinking experiments. Attempts to reconcile this discrepancy have been made in the recent reviews by Ifuku (2015). High-resolution structural analysis is required to reveal the detailed extrinsic lumenal relationships in the PSII-LHCII supercomplex.

## Other Eukaryotic Algae

### Red Algae

**FIGURE 5:**
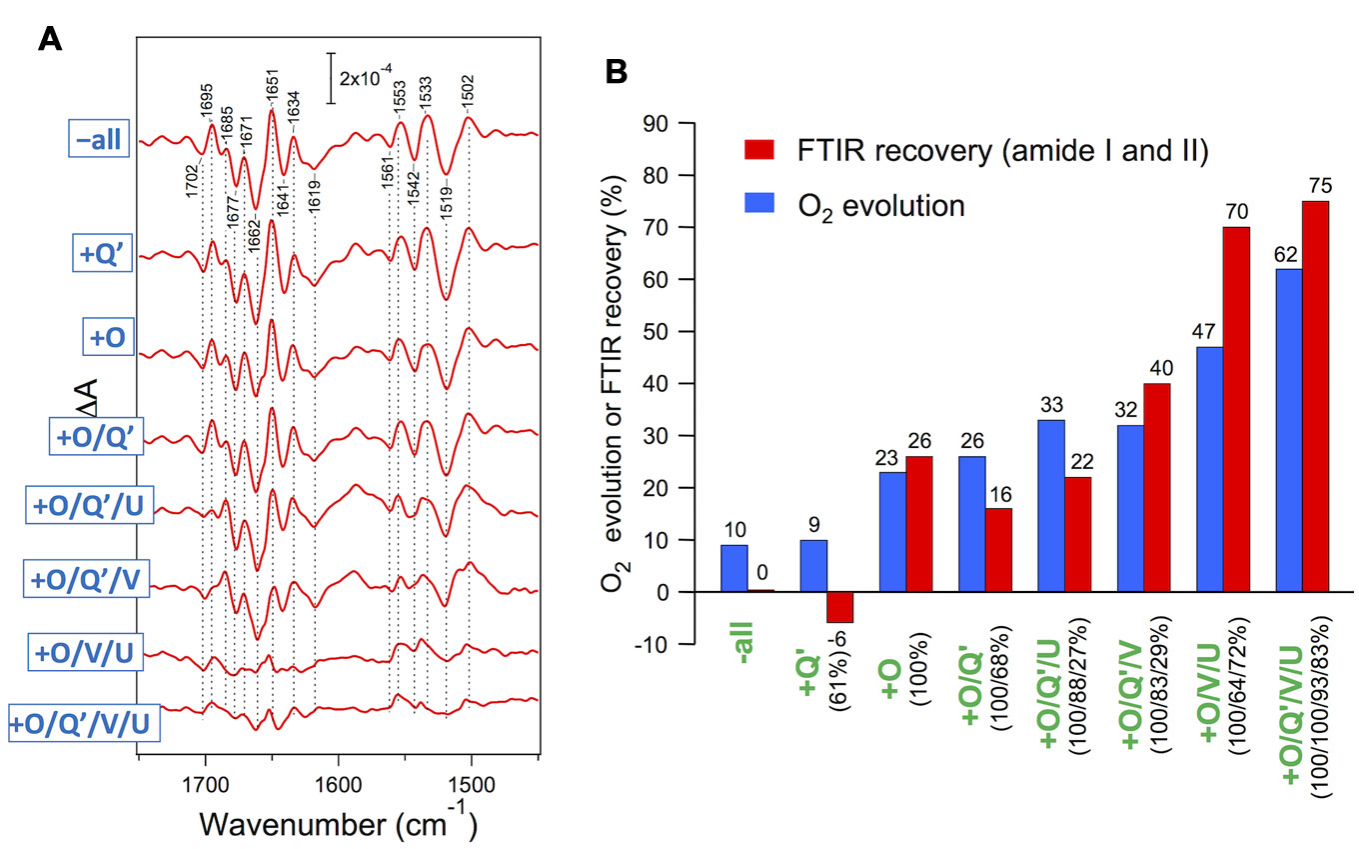
**(A)** Recovery of amide I and II bands in FTIR spectra upon reconstitution of PSII core complexes of *Cyanidium caldarium* with extrinsic proteins. Double difference spectra between the S_2_-minus-S_1_ FTIR difference spectra of reconstituted and untreated PSII samples are shown. The samples involved 10 mM CaCl_2_. **(B)** Comparison of the recovery of the FTIR amide I and II bands (red bars) with that of O_2_ evolution (blue bars: taken from Enami et al., 1998) upon reconstitution with extrinsic proteins. The amounts of reconstituted extrinsic proteins are indicated as figures in parentheses. O_2_ evolution was measured in the presence of 50 mM CaCl_2_ (Enami et al., 1998). Reproduced from Uno et al. (2013).

Red algae have PsbV, PsbU, and PsbQ′ in addition to the common PsbO. Enami et al. (1998) performed reconstitution of the PSII core complexes of *Cyanidium caldarium* with these extrinsic proteins in various combinations. PsbO and PsbQ′ were independently bound to PSII, whereas PsbV and PsbU required binding of PsbO and could fully bind only in the presence of both PsbO and PsbQ′. The O_2_-evolution activity was recovered step by step in the course of reconstitution (**Figure 5B**, blue bars). Uno et al. (2013) examined the effect of rebinding the extrinsic proteins to the core complexes of *C. caldarium* on the OEC protein conformation by S_2_-minus-S_1_ FTIR measurements. The vibrations of carboxylate groups were virtually unchanged even by removal of all extrinsic proteins, indicating that the interactions of the Mn_4_CaO_5_ cluster were not affected by extrinsic proteins. By contrast, amide I and II bands, reflecting changes in polypeptide main chains, were significantly altered by the removal and rebinding of extrinsic proteins (**Figure 5A**). The extent of the recovery of the amide I and II bands by reconstitution with extrinsic proteins (**Figure 5B**, red bars) correlated well with that of O_2_-evolution activity (blue bars), revealing a direct relationship of the protein conformation of the OEC with its activity. It was shown that PsbV binding mainly contributed to the restoration of the protein conformation of the OEC, resembling the function of PsbP in higher plants. PsbU seemed to support the proper binding of PsbV, which is analogous to PsbQ in higher plants. These facts support a model proposed by Ido et al. (2014), in which PsbP in higher plant PSII occupies a position roughly similar to that occupied by PsbV in the cyanobacterial crystal structure (Ifuku, 2015). Crystallization and preliminary X-ray diffraction analysis have already been reported (Adachi et al., 2009), so that the structure of red algal PSII will be available in the near future.

### Diatoms

Diatom PSII contains a fifth extrinsic protein, Psb31, in addition to the four red algal-type extrinsic proteins. Psb31 is suggested to have originated via a secondary endosymbiosis event (Nagao et al., 2013). Reconstitution experiments using proteins from a centric diatom, *Chaetoceros gracilis*, have suggested that Psb31 binds directly to PSII intrinsic proteins. Noticeably, PSII reconstituted with Psb31 alone can partially restore oxygen-evolving activity in the absence of PsbO, suggesting that Psb31 has a novel and specific function in diatom PSII. Analysis of the crystal structure of Psb31 has revealed a four-helix bundled structure showing partial structural similarity with PsbQ family proteins (Nagao et al., 2013). Thus, two copies of PsbQ-like proteins with different binding and functional properties seem to be present as extrinsic subunits in the diatom PSII. A further study using FTIR is required to elucidate the structural coupling of those extrinsic proteins with the OEC in diatoms. Crystallization and preliminary X-ray diffraction analysis of the diatom PSII complex have not yet been reported.

## Cyanobacteria

### Molecular Functions of Extrinsic Proteins, Briefly

The crystal structure of PSII from *Thermosynechococcus vulcanus* (Umena et al., 2011) shows the location and interaction of cyanobacterial PSII extrinsic subunits PsbO, PsbU, and PsbV on the lumenal surface of PSII. Each extrinsic protein has multiple interactions with both membrane-intrinsic and -extrinsic subunits of PSII, affecting the stabilization of the complex as a whole. In brief, PsbO interacts with CP43, CP47, D1, D2, and PsbU subunits, PsbU interacts with CP47, PsbO, and PsbV, and PsbV interacts with CP43, D1, D2, and PsbU. Detailed information about the amino acid residues involved in these interactions have been summarized by Bricker et al. (2012). Although none of these proteins provide a ligand to the catalytic Mn_4_CaO_5_ cluster, they have critical roles: they protect the metal cluster from reductants outside the PSII complex and optimize the required ionic environments, such as those of Ca^2+^ and Cl^-^, in the OEC. The functions of each PSII extrinsic subunit have been intensively studied by deletion mutagenesis of cyanobacterial cells (Seidler, 1996; Bricker et al., 2012). It is of note that crystal structures and theoretical calculations suggest another role of the extrinsic proteins — namely, the maintenance of access channels for substrate water to the Mn_4_CaO_5_ cluster and exit channels for the products (molecular oxygen and protons; Linke and Ho, 2014; Vogt et al., 2015). A number of amino acid residues in the extrinsic proteins have been predicted to be associated with these channels, but their actual roles still need to be investigated experimentally.

### Structural Coupling with the OEC

**FIGURE 6:**
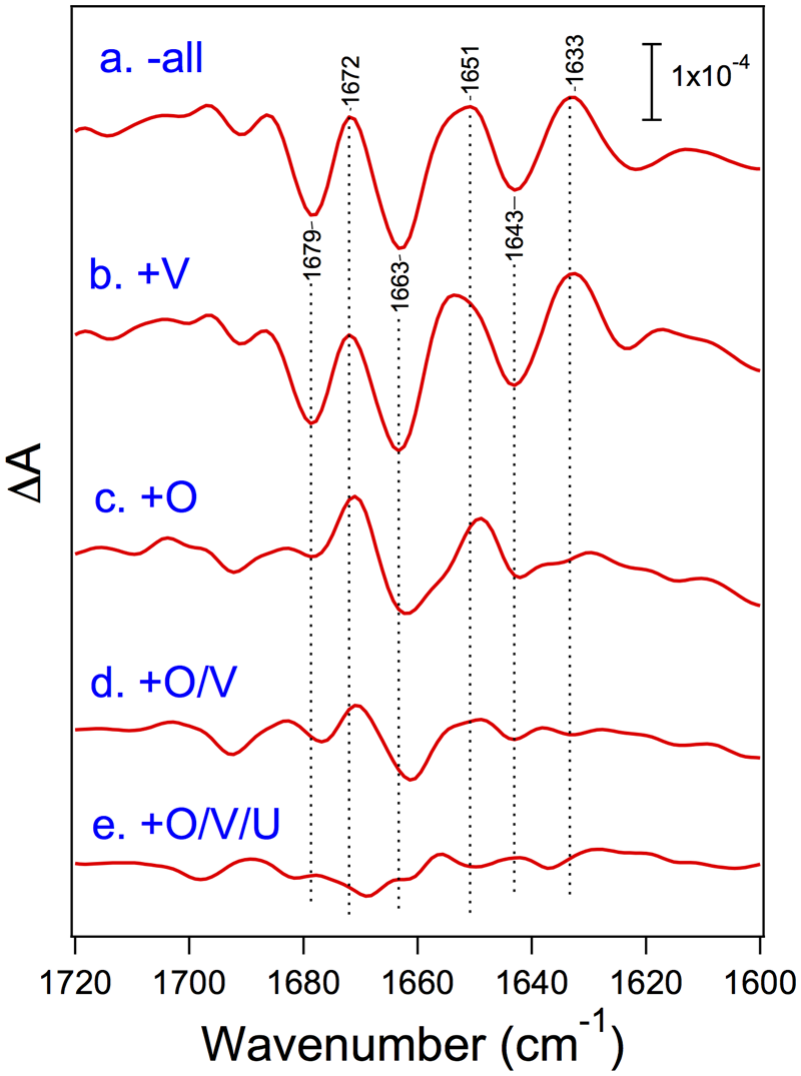
**Amide I region of the untreated-minus-treated double-difference spectra of the S_2_-minus-S_1_ difference spectra of the PSII core complexes from cyanobacterium *Thermosynechococcus elongatus***. PSII core complexes were depleted of all the extrinsic proteins **(a)** and then reconstituted with PsbV **(b)**, PsbO **(c)**, PsbO/V **(d)**, and PsbO/V/U **(e)**. The samples involved 100 mM CaCl_2_. Reproduced from Nagao et al. (2015).

Nagao et al. (2015) recently reported the FTIR measurements of S_2_-minus-S_1_ difference spectra using PSII core complexes from *T. elongatus* reconstituted with its extrinsic proteins, PsbO, PsbV, and PsbU. Under the condition of low-concentration CaCl_2_ (5 mM), the spectral intensity was mostly lost when all the extrinsic proteins were removed, whereas the intensity in the carboxylate region was fully recovered by the binding of PsbO, revealing the significant role of PsbO in stabilizing the Mn_4_CaO_5_ cluster at a low CaCl_2_ concentration. Even at a high CaCl_2_ concentration (100 mM), amide I bands were significantly affected by removal of all the extrinsic proteins, indicative of the protein conformational changes in the OEC (**Figure 6**). The bands largely recovered when PsbO was bound, and further stepwise recoveries were observed by the binding of PsbV and then PsbU. Thus, in cyanobacteria, the binding of PsbO seems mainly to determine the conformation of the OEC. PsbV and PsbU induced the recovery of specific amide I bands, and hence it was suggested that these extrinsic proteins affected different regions of polypeptide chains from those affected by PsbO. PsbO and PsbV have multiple interactions with different membrane-intrinsic subunits of PSII. This makes it difficult to assign the exact polypeptide regions affected by PsbO and PsbV during the S_1_-to-S_2_ transition. Nevertheless, the recoveries of the OEC protein conformation were very consistent with those of O_2_-evolution activity upon reconstitution with extrinsic proteins reported by Shen and Inoue (1993), suggesting again the correlation between the OEC activity and its protein conformation.

### CyanoP and CyanoQ

It has been known that cyanobacteria have ancestral homologs of eukaryotic PsbP and PsbQ, CyanoP, and CyanoQ (De Las Rivas et al., 2004). Their involvement in Ca^2+^ and Cl^-^ retention has been suggested (Thornton et al., 2004; Aoi et al., 2014), however, they are not included in current crystal structures of PSII from thermophilic cyanobacteria (Umena et al., 2011). CyanoQ is reported to bind PSII tightly and to optimize oxygen evolution (Roose et al., 2007a), and a recent study using chemical cross-linking suggests that CyanoQ is closely associated with PsbO and CP47 proteins in *Synechocystis* sp. PCC 6803 (Liu et al., 2014). Furthermore, two molecules of CyanoQ may interact at the interface of the PSII dimers, indicating that CyanoQ, together with PsbO, is important for PSII dimer stability (Liu et al., 2014). Binding of multiple CyanoQ copies to the PSII assembly intermediates without PsbU and PsbV was recently reported (Liu et al., 2015). Similarly, interaction of CyanoP with the binding site in PSII, which is occupied by the PsbO subunit in mature PSII complexes, was suggested (Cormann et al., 2014). These facts suggest that localizations of CyanoP and CyanoQ in cyanobacterial PSII would be largely different form those of PsbP and PsbQ in higher plant PSII. It is likely that both CyanoP and CyanoQ have auxiliary functions in regulating and stabilizing the association of the extrinsic subunits with the PSII core.

## Functional Changes During Evolution

**FIGURE 7:**
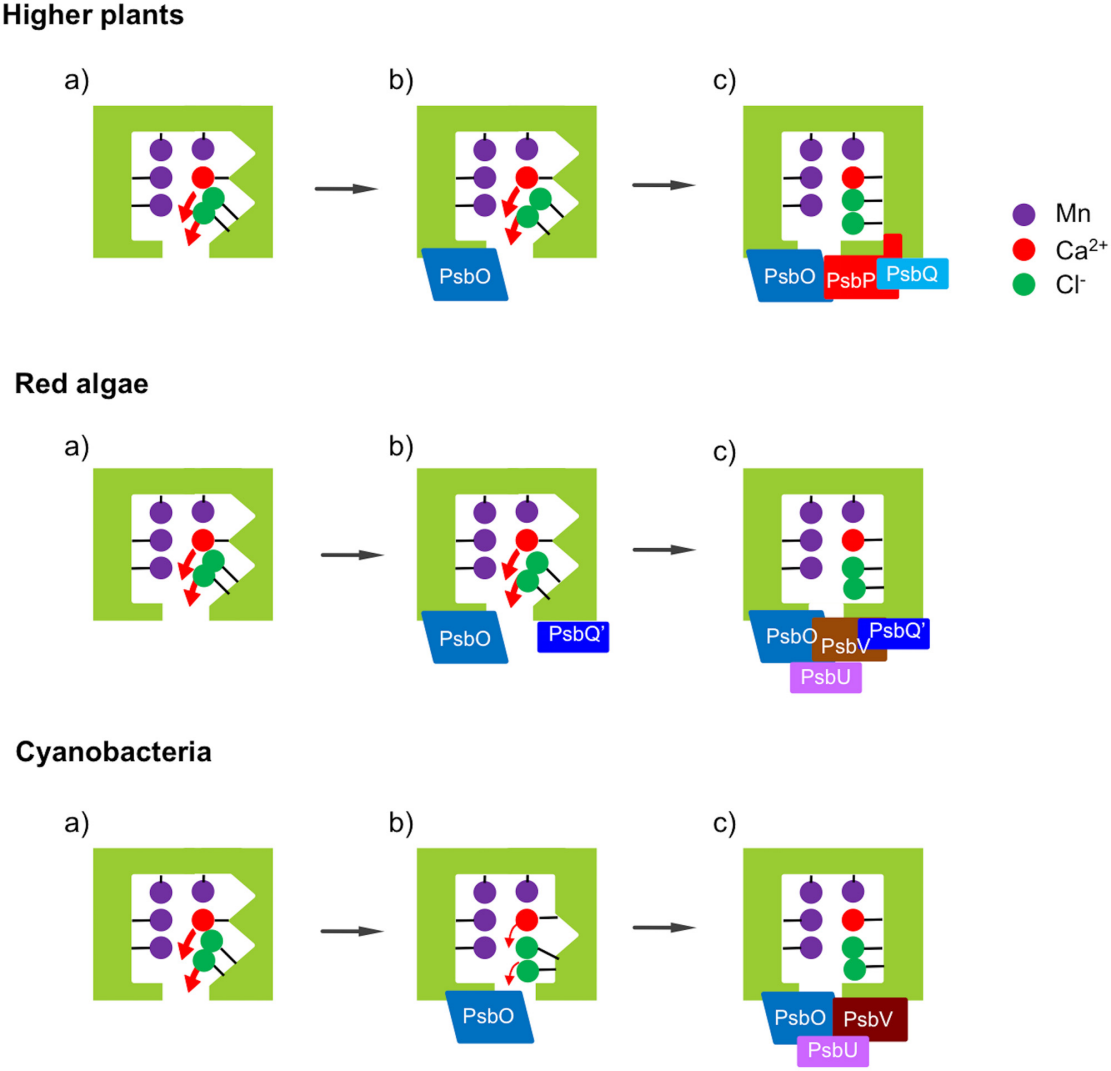
**Schematic view of the effects of the extrinsic proteins on the protein conformation of the OEC in different photosynthetic organisms. (a)** Upon depletion of all the extrinsic proteins, the protein conformation of the OEC is significantly altered. **(b)** Binding of PsbO does not restore the structure of the OEC in higher plants and red algae, but largely restores it in cyanobacteria. **(c)** Further binding of PsbP can restore the structure of the OEC mostly in higher plants, while both PsbV and PsbU are required for full recovery in red algae and cyanobacteria. PsbQ and PsbQ′ function to stabilize the binding of PsbP and PsbV, respectively.

Cyanobacteria are thought to be an ancestor of chloroplasts in both the red lineage involving red algae and diatoms and the green lineage involving green algae and higher plants. Reconstitution and FTIR studies mentioned above showed that PsbO, PsbV, and PsbP are mainly responsible for determining the protein conformation of the OEC in cyanobacteria (Nagao et al., 2015), red algae (Uno et al., 2013), and higher plants (Tomita et al., 2009), respectively. The FTIR results obtained so far are summarized in a schematic diagram of the relationship between the OEC conformation and the binding of the extrinsic proteins (**Figure 7**). The protein conformation of the OEC detected by FTIR amide I bands directly correlated with O_2_-evolution activity in all the cases. It probably regulates the binding of Ca^2+^ and Cl^-^ ions in the OEC by changing the dissociation constants and/or the energy barrier in releasing and binding reactions.

It is interesting that although PsbO was conserved in all oxyphototrophs, its function was not fully conserved and the function of regulating the OEC conformation was transferred to PsbV and PsbP in the red and green lineages, respectively, during evolution. This suggests that PsbP and PsbQ did not simply replace the functions of PsbV and PsbU, necessitating a substantial reconsideration of historical concepts regarding the evolution of the extrinsic subunits of PSII. PsbO undoubtedly keeps the important function of stabilizing the Mn_4_CaO_5_ cluster in all species. It is possible that PsbV or PsbP took over the specific function of the regulation of protein conformations from PsbO to realize a more delicate control over the O_2_-evolving reaction without the destruction of the Mn_4_CaO_5_ cluster. By contrast, the function of PsbU in cyanobacteria as supporting the binding of PsbV seems to remain in red algae but be transferred to PsbQ, which stabilizes the binding of PsbP, in higher plants (Kakiuchi et al., 2012). It has been reported that CyanoQ in cyanobacterial PSII also stabilizes the PsbV binding to PSII, thereby contributing to the protection of the catalytic Mn cluster of the OEC (Kashino et al., 2006). Interestingly, PsbQ′ in red algal PSII is required for the stable binding of PsbV (Enami et al., 1998). Presumably, the molecular functions of CyanoQ, PsbQ′, and PsbQ are partly conserved during evolution, while the interacting partner has changed from PsbV to PsbP. Further studies will reveal how the variations of PSII extrinsic proteins confer functional differences to PSII, presumably by fine tuning of the light-induced charge separation and the OEC reactivity, and how they affect the processes of the assembly and repair of the entire PSII complex.

## Author Contributions

All authors listed, have made substantial, direct and intellectual contribution to the work, and approved it for publication.

## Conflict of Interest Statement

The authors declare that the research was conducted in the absence of any commercial or financial relationships that could be construed as a potential conflict of interest.
